# Cost‐Effectiveness Analysis of Patient Self‐Management of Warfarin Among Patients With Non‐Valvular Atrial Fibrillation

**DOI:** 10.1002/phar.70154

**Published:** 2026-05-14

**Authors:** Warittakorn Kategeaw, Daniel M. Witt, Jordan B. King, Daniel C. Malone, Nathorn Chaiyakunapruk

**Affiliations:** ^1^ Department of Pharmacotherapy, College of Pharmacy University of Utah Salt Lake City Utah USA; ^2^ Department of Population Health Sciences, Spencer Fox Eccles School of Medicine University of Utah Salt Lake City Utah USA; ^3^ Institute for Health Research Kaiser Permanente Colorado Aurora Colorado USA; ^4^ IDEAS Center Veterans Affairs Salt Lake City Healthcare System Salt Lake City Utah USA

**Keywords:** anticoagulation, cost‐effectiveness, non‐valvular atrial fibrillation, self‐management, warfarin

## Abstract

**Background:**

Patient self‐management (PSM) is a warfarin management strategy that allows patients to independently adjust their doses. PSM significantly reduces thrombosis events and has been widely implemented internationally. However, adoption of PSM is limited in the United States. This study estimated the cost‐effectiveness of PSM compared with available strategies, including anticoagulation management services (AMS) and usual care (UC) for patients with non‐valvular atrial fibrillation (NVAF) aged 70 years and older in the United States.

**Methods:**

A cost‐effectiveness analysis was performed to estimate lifetime costs and outcomes of PSM compared with AMS and UC employing a Markov model. The model simulated a 70‐year‐old with NVAF over the patient's lifetime from modified societal and payer perspectives. Transition probabilities were derived from annual event rates reported in the ARISTOTLE trial. Comparative efficacy and safety among the interventions were obtained from a network meta‐analysis. Costs, presented in 2024 US dollars (USD), and utility data were obtained from data sources in the United States. A discount rate of 3% was applied for future costs and outcomes. One‐way sensitivity analysis and probabilistic sensitivity analysis were performed to assess the robustness of the findings.

**Results:**

The lifetime costs from the modified societal perspective were $137,509 USD for UC, $129,216 USD for AMS, and $76,515 USD for PSM. Discounted quality‐adjusted life years (QALYs) were estimated to be 8.27 for UC, 8.30 for AMS, and 8.47 for PSM. Additionally, PSM substantially reduced stroke incidence compared with AMS and UC, contributing to lower costs and greater QALYs. Probabilistic sensitivity analysis showed that PSM appeared to be the most cost‐effective at any willingness‐to‐pay threshold.

**Conclusions:**

PSM is the most cost‐effective strategy for managing warfarin in the target population at any willingness‐to‐pay threshold. Implementation of PSM in the US health care setting should be considered.

## 1 Introduction

Despite the declining trend in its overall use, warfarin has been a reliable and essential anticoagulant for decades because of its effectiveness and affordability [1]. However, its use is challenging because of a narrow therapeutic index, slow onset and offset, high discontinuation rates, and variable patient response [2]. In addition, warfarin has several known drug–drug and diet‐drug interactions, making careful management essential [2, 3]. Regular laboratory monitoring of anticoagulation is required especially during initiation, medication changes, or dosage adjustments [4]. Traditionally, this monitoring takes place in health care facilities, with clinicians managing dose changes. Although the traditional monitoring model is safe and effective, it can be inconvenient for patients and burdensome to health care systems.

Patient self‐management (PSM) provides an alternative approach that empowers patients to monitor their own laboratory values and adjust warfarin doses when needed [5, 6]. Compared to other care models, PSM is linked to significantly lower rates of thromboembolism, greater patient satisfaction, improved engagement and independence, and higher efficiency [7, 8]. In particular, PSM involves more frequent monitoring without the burden of travel, allowing patients to maintain better control over their treatment, reflected in a higher time in therapeutic range (TTR) [8]. Evidence‐based guidelines recommend PSM over traditional strategies, including anticoagulation management services (AMS). The 2018 American College of Chest Physicians Guidelines highlight benefits such as convenience, autonomy, empowerment, and enhanced quality of life [9]. Similarly, the American Society of Hematology 2018 guidelines strongly recommend PSM for managing venous thromboembolism (VTE) [10].

Although PSM is widely adopted in European countries, such as Germany, the United Kingdom, and Spain, it remains virtually unused in the United States [7, 11, 12, 13, 14]. US clinicians are generally less familiar with the supporting evidence and often unconvinced of its advantages over clinician‐managed care [15]. Although clinical feasibility and efficacy have been demonstrated, the economic impact of implementing PSM in the United States is unknown [16]. Establishing its cost‐effectiveness could increase clinician willingness to offer this evidence‐based care model. In this study, we conducted a cost‐effectiveness analysis comparing PSM with traditional warfarin management through AMS and usual physician care (UC) in patients with non‐valvular atrial fibrillation (NVAF), the most common indication for anticoagulation therapy.

## 2 Methods

A health economic analysis plan was developed prior to conducting the analysis and is available at https://doi.org/10.17605/OSF.IO/8SMUJ. Before conducting the analysis, we held a stakeholder engagement session with patients, clinicians, and both institutional‐ and state‐level decision makers to refine the approach. Based on their feedback, we added an AMS comparator group alongside UC to reflect that many warfarin patients are managed in AMS. In this study, AMS refers to care provided by dedicated anticoagulation clinicians within a structured program, whereas UC refers to care delivered by prescribing clinicians outside an AMS. We also incorporated the payer perspective and removed myocardial infarction as an outcome because it is not a primary indication for warfarin in NVAF. The study population focuses only on patients on warfarin. We assumed no change in medication (i.e., transition to direct oral anticoagulants (DOACs) was not allowed). This work follows the Consolidated Health Economic Evaluation Reporting Standard 2022 (CHEERS 2022) [17] (Table S1).

### 2.1 Decision Model

**FIGURE 1:**
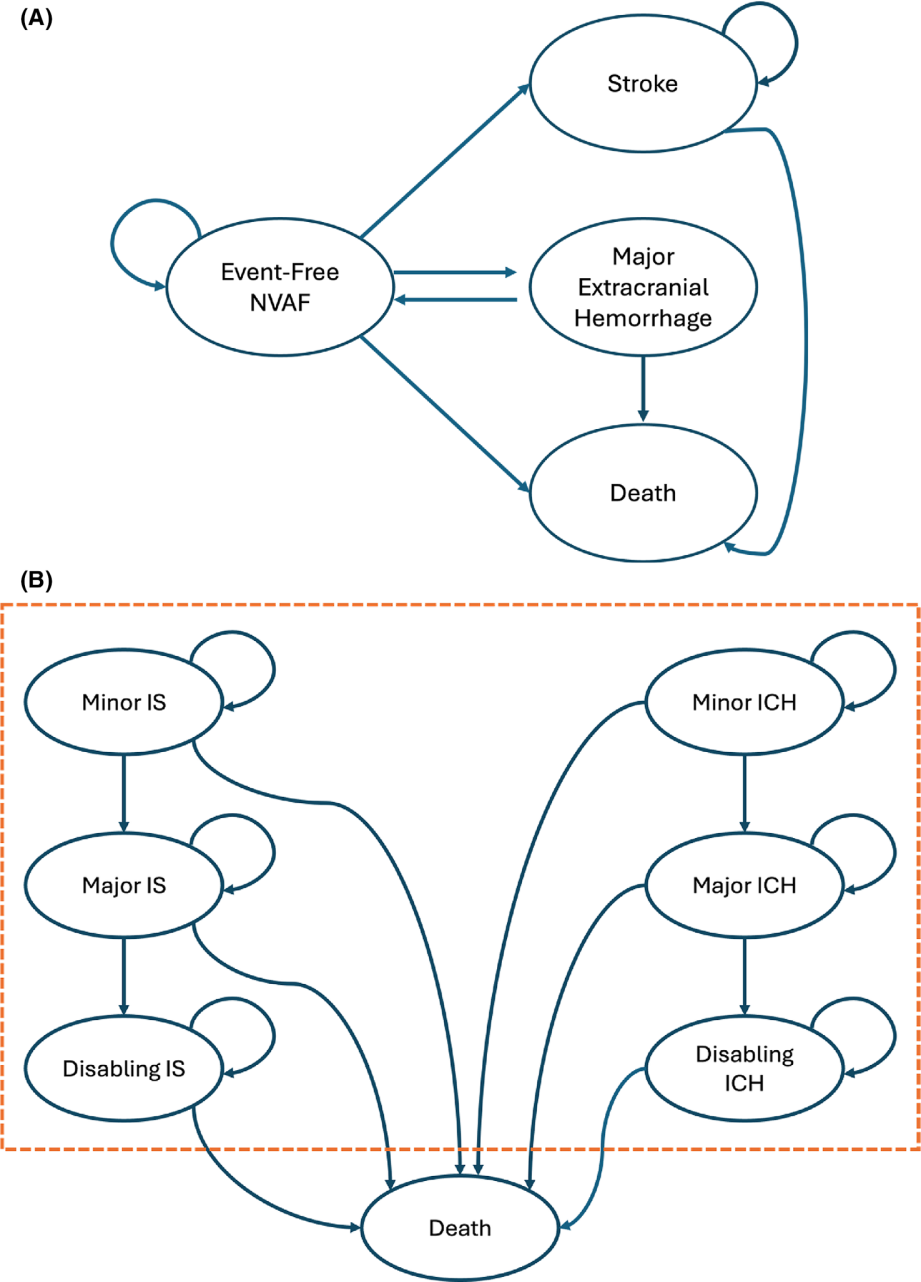
(A) Simplified decision model structure. (B) Simplified decision model in Stroke health state. ICH, Intracranial haemorrhage; IS, Ischemic stroke; NVAF, Non‐valvular atrial fibrillation.

The Markov model used in this study was adapted from prior cost‐effectiveness analyses [18, 19]. Due to limited data on relative risks across interventions, ischemic stroke and intracranial hemorrhage were combined into a single health state, “Stroke”. The model was built in Microsoft Excel (Microsoft, Washington, USA) and simulated US patients aged 70 years or older with NVAF receiving warfarin for stroke prevention. Patients entered the model without prior anticoagulation‐related events, and clinical events were treated as mutually exclusive (Figure 1A,B). Each 3 month cycle allowed for stroke, major extracranial hemorrhage, or death. Stroke events were classified as ischemic or hemorrhagic and further categorized by severity: minor, major without disability, and major with disability. After an ischemic stroke or intracranial hemorrhage, patients could not experience other event types; they either remained in their current state or progressed to a more severe health state associated with the recurrence. In contrast, patients with non‐fatal major extracranial hemorrhage could return to the “healthy with NVAF” state and are at risk for future events. Major extracranial hemorrhage was defined according to the International Society on Thrombosis and Hemostasis (ISTH) [20, 21]. Intervention effects were assumed to occur immediately and remain constant over time.

### 2.2 Model Parameters

#### 2.2.1 Transition Probability

Transition probabilities for UC patients were derived from the incidence rates reported in the ARISTOTLE trial [21]. Probabilities for AMS and PSM were adjusted by applying relative risks versus UC from a network meta‐analysis of 8100 participants across 28 trials [8]. Stroke events were further divided into ischemic stroke and intracranial hemorrhage based on subtype proportions [22]. Event severity distributions were obtained from observational studies [23, 24, 25]. Severity varied by health state. Recurrent stroke probabilities were assumed to be independent of treatment type [26, 27]. Baseline mortality was based on age‐specific rates from the Centers for Disease Control and Prevention (CDC) and adjusted for the increased risk associated with atrial fibrillation (Table 1) [28, 29, 30, 31, 32].

**TABLE 1:** Base‐case values, ranges, and distributions of model inputs (transition probability).

Parameters	Base case	Range/SE	Distribution	Source (s)
Annual event rate				
Stroke	1.15%	1.33%–1.71%	Gamma	Granger 2011 [21]
Major extracranial hemorrhage	2.27%	1.89%–2.69%	Gamma	Granger 2011 [21]
Recurrent stroke event	2.72%	1.68%–4.01%	Gamma	Easton 2012 [26] Dorian 2014 [27]
Proportion of severity per clinical event				
Ischemic stroke				
Minor	0.425	NA	Fixed	Adjusted from Hankey 2002 [23]
Major	0.366	NA	Fixed	
Disabled	0.127	NA	Fixed	
Fatal	0.082	NA	Fixed	
Intracranial hemorrhage				
Minor	0.2	NA	Fixed	Hylek 1994 [24], Lee 2012 [25]
Major	0.15	NA	Fixed	
Disabled	0.1	NA	Fixed	
Fatal	0.53	NA	Fixed	
Major extracranial hemorrhage, fatal	0.0147	NA	Fixed	
Event‐rate adjustments				
Increased risk of death given an event				
Atrial fibrillation	1.3	1.0–1.5	Log‐normal	Freeman 2011 [28] Yuan 1998 [29], Wyse 2001 [30], Dries 1998 [31]
Relative risks of stroke compared with usual care				
Usual care	1	NA	NA	Dhippayom 2024 [8]
Anticoagulation clinic	0.95	0.10–8.94	Log‐normal	Dhippayom 2024 [8]
Patient self‐management	0.5	0.10–2.44	Log‐normal	Dhippayom 2024 [8]
Relative risks of major extracranial hemorrhage compared with usual care				
Usual care	1	NA	NA	Dhippayom 2024 [8]
Anticoagulation clinic	0.89	0.48–1.68	Log‐normal	Dhippayom 2024 [8]
Patient self‐management	0.98	0.65–1.46	Log‐normal	Dhippayom 2024 [8]

Abbreviations: NA, Not Available; SE, Standard Error.

#### 2.2.2 Utilities

Patient utilities for each health state were obtained from published sources (Table 2). The baseline utility for US patients with NVAF is 0.81 [33]. Disutilities for clinical events were applied by subtracting from the baseline value, with adjustments based on event type and severity [34, 35, 36, 50]. Neurological events, including ischemic stroke and intracranial hemorrhage, resulted in permanent utility decrements, whereas major extracranial bleeding reduced utility for only 2 weeks. After recovery, patients returned to the NVAF utility for the remainder of the cycle. Utilities were assumed to be similar across all interventions.

**TABLE 2:** Base‐case values, ranges, and distributions of model inputs (utilities and costs).

Parameters	Base case	Range/SE	Distribution	Source (s)
Utility/disutility				
NVAF	0.81	0.68–0.91	Beta	Sullivan 2006 [33]
Neurological event				
Minor	−0.29	−0.30 to −0.28	Beta	Harrington 2013 [34], Sullivan 2005 [35]
Major	−0.45	−0.46 to –0.43	Beta	Harrington 2013 [34], Sullivan 2005 [35]
Disabled	−0.45	−0.46 to −0.43	Beta	Harrington 2013 [34], Sullivan 2005 [35]
Major extracranial bleeding, 2 weeks	−0.181	−0.114 to −0.106		Sullivan 2006 [36] Sullivan 2005 [35]
Costs				
Anticoagulation management cost, USD				
Warfarin (not including INR monitoring), per day	0.67	0.17–0.73	Gamma	RedBook [37]
Cost of INR laboratory	8.91	5.94–14.85	Gamma	CMS [38]
Anticoagulation service cost (per visit)	11.52	10.58–15.10	Gamma	CMS [39]
Usual care cost (per visit)	36.68	33.85–49.04	Gamma	CMS [39]
Frequency of clinic visits				
UC, first 2 months	3 visits per month		Fixed	Expert opinion
UC, month 3+	1 visit per 2 months		Fixed	
AMS, first 2 weeks	2 visits per week		Fixed	
AMS, week 3 to month 2	2 visits per month		Fixed	
AMS, month 3–6	3 visits per 2 months		Fixed	
AMS, after month 6 (per month)	5 times per 6 months		Fixed	
PSM‐related costs, USD				
PSM patients training session	124.44	86.52–162.16	Gamma	BLS [40]
Device for PSM (CoaguChek XS)	595	595.00–1100.00	Gamma	Medical Device Depot, Medical Rite, Healthcare Supply Pros [41, 42, 43]
INR strip (per item)	5.6	5.1–8.83	Gamma	
Lancet (per item)	0.11	0.03–0.13	Gamma	
CoaguChek XS Softclix	9.99	7.34–18.00	Gamma	
Event cost, USD				
Ischemic Stroke				
Minor	10,348	10,162–10,535	Gamma	Adjusted from HCUP 2022 [44]
Major	13,362	13,164–13,561	Gamma	
Disabled	22,929	22,451–23,428	Gamma	
Intracranial hemorrhage				
Minor	10,348	10,162–10,535	Gamma	Adjusted from HCUP 2022 [44]
Major	13,362	13,164–13,561	Gamma	
Disabled	22,929	22,451–23,428	Gamma	
Major extracranial hemorrhage	13,682	13,551–13,812	Gamma	Adjusted from HCUP 2022 [44]
Maintenance costs (per 3 months), USD				
Ischemic Stroke				
Minor	8851	6815–10,883	Gamma	Adjusted from Yousufuddin 2020 [45]
Major	28,006	19,201–36,807	Gamma	
Disabled	51,838	35,070–68,608	Gamma	
Intracranial hemorrhage				
Minor	17,448	12,033–22,863	Gamma	Adjusted from Yousufuddin 2020 [45]
Major	81,255	48,145–114,281	Gamma	
Disabled	27,239	16,378–35,701	Gamma	
Discount rate	0.03	0.01–0.05	Beta	
Average distance to hospital, miles	8.7	IQR = 14.52	Gamma	HCUP 2021 [46]
Mileage rate, USD	0.18	NA	NA	Internal Revenue Service 2020 [47]
Number of hours per week of informal care for stroke				
Minor	19	15.2–22.8	Gamma	Ganapathy 2015 [48]
Major	38	30.4–45.6	Gamma	
Disabled	49	39.2–58.8	Gamma	
Hourly rate	23.8	14.42–60.44	Gamma	[49]

Abbreviations: AMS, Anticoagulation Management Services; BLS, U.S. Bureau of Labor Statistics; CMS, Center for Medicaid & Medicare Services; HCUP, Healthcare Cost and Utilization Project; INR, International Normalized Ratio; IQR, Interquartile Range; NA, Not Available; NVAF, Non‐Valvular Atrial Fibrillation; PSM, Patient Self‐Management; SE, Standard Error; UC, Usual Care; USD, United States Dollar.

#### 2.2.3 Costs

The model included direct medical, non‐medical costs, and indirect costs accounting for the caregiver's opportunity cost (Table 2). However, we did not incorporate productivity loss from our target population, as the US NVAF population is generally of retirement age. According to the Bureau of Labor Statistics (BLS), the civilian labor force participation rate of those who are 65 to 74 years is 27.1% in 2024, and declined to 8.6% in those who are 75 years and older [51]. Individuals aged 65 years and older accounted for 7.0% of the labor force in 2024 [52]. Given the demographic profile, patient productivity loss is expected to be minimal. All costs were adjusted to 2024 US dollar (USD) values using the Consumer Price Index (CPI) [53].

Direct medical costs comprised drug acquisition, event management, and maintenance. Warfarin's wholesale acquisition cost ($0.67 per 5‐mg tablet) was applied [37]. PSM‐related costs included a portable international normalized ratio (INR) monitoring system (CoaguChek XS system (Indianapolis, IN, USA)), INR strips, lancets, and a fingerstick device (CoaguChek XS Softclix (Indianapolis, IN, USA)) with prices sourced online [41, 42, 43]. Devices were assumed to last 5 years, and one strip and lancet were used per test. Event management costs, classified by Medicare Severity Diagnosis‐Related Group (MS‐DRG), were obtained from the National Inpatient Survey (NIS) under the Healthcare Cost and Utilization Project (HCUP), which contains all‐payer hospital data [44]. Event management costs were applied only to patients who developed a new event during transitions between health states in the simulation. Post‐event maintenance costs were drawn from published cost‐effectiveness analyses [45]. Maintenance costs were applied to all individuals residing in each health state.

Clinic visits and INR monitoring varied by intervention, reflecting real‐world patterns. As summarized in Table 2, UC patients were assumed to visit the clinic three times per month for 2 months, then every 2 months. AMS patients visited twice weekly for 2 weeks, then every 2 weeks until month 2, followed by three visits every 2 months (months 3–6) and five times per 6 months thereafter. PSM patients followed AMS visit patterns during training, then transitioned to weekly INR testing at home, assuming stable control. The frequency of clinic visits was assumed based on expert opinion to reflect real‐world monitoring intensity, which often exceeds the minimum requirements suggested by clinical guidelines. The management costs were calculated using (Equation (1)) on a 3‐month cycle unit basis.

(1) $$\begin{array}{c} \text{Management cost}=\left(\text{Frequency}\times \text{Unit Cost}\;\mathrm{per}\;\text{Visit}\right)\\ \;+\mathrm{PSM}-\text{related cost}\;\left(\text{if applicable}\right) \end{array}$$

Direct non‐medical costs included patient transportation, calculated using the average hospital distance from HCUP and the Internal Revenue Service (IRS) standard mileage rate [46, 47].

Indirect cost, including the caregiver's opportunity costs, was applied to long‐term care for stroke, characterized by stroke severity. Caregiver's opportunity costs were calculated based on the time required for informal care valued at the national hourly wage rate [48, 49].

### 2.3 Analytical Methods

#### 2.3.1 Base‐Case Analysis

We evaluated costs and quality‐adjusted life years (QALYs) over a lifetime horizon from a modified societal perspective. A secondary analysis from the payer perspective excluded direct non‐medical costs. Both costs and outcomes were discounted at 3% per year [54]. Incremental cost‐effectiveness ratios (ICERs) were calculated using mean costs and QALYs [55]. A willingness‐to‐pay threshold of $100,000 USD per QALY gained was applied.

#### 2.3.2 Sensitivity Analysis

Sensitivity analyses were conducted to assess the robustness of the model outputs to uncertainty in input parameters. One‐way sensitivity analyses varied individual parameters to evaluate their impact on the net monetary benefit (NMB) of PSM. Additionally, a probabilistic sensitivity analysis was performed by sampling each parameter across its plausible range. Gamma distributions were applied to event rates and costs, beta distributions to utilities, and log‐normal distributions to multiplier parameters.

## 3 Results

### 3.1 Base‐Case Analysis

The lifetime outcomes from each intervention, including life years, QALYs, and lifetime costs for the base case, are summarized in Table 3. From a modified societal perspective, the lifetime costs were $137,509 USD for UC, $129,216 USD for AMS, and $76,515 USD for PSM. Discounted QALYs were 8.27 for UC, 8.30 for AMS, and 8.47 for PSM. From the payer perspective, lifetime costs were $103,656 USD for UC, $96,941 USD for AMS, and $59,050 USD for PSM, with discounted QALYs unchanged.

**TABLE 3:** Projected life years, lifetime quality‐adjusted life years (QALY), and lifetime costs from modified societal and payer perspectives.

Intervention	Discounted life years	Discounted QALY	Discounted costs ($2024)	Incremental QALY	Incremental costs
Modified societal perspective					
Usual care	10.66	8.27	137,509	Reference	
Anticoagulation management service	10.66	8.30	129,216	0.02	−8292
Patient self‐management	10.68	8.47	76,515	0.17	−52,701
Payer perspective					
Usual care	10.66	8.28	103,656	Reference	
Anticoagulation management service	10.66	8.30	96,941	0.02	−6712
Patient self‐management	10.68	8.47	59,050	0.17	−37,889

### 3.2 Sensitivity Analysis

Probabilistic sensitivity analyses were conducted (Figure S1A). Compared with UC, 77.2% of 1000 iterations indicated that AMS achieved better outcomes at lower costs. Other scenarios also occurred when comparing AMS with UC: 6% of iterations showed lower costs but worse outcomes, and 22.2% showed higher costs and worse outcomes (Figure S1B). In contrast, 89.4% of iterations for PSM demonstrated better outcomes at lower costs compared with UC (Figure S1C). When comparing PSM to AMS, PSM provided better outcomes at lower costs in 52.8% of iterations (Figure S1D).

The cost‐effectiveness acceptability curve (Figure S2) indicated that PSM was the most likely to be cost‐effective across all willingness‐to‐pay thresholds, with probabilities ranging from 51.5% to 52.6%. The AMS strategy was consistently cost‐effective compared with UC at every threshold. At a willingness‐to‐pay threshold of $100,000 per QALY, the probabilities of being cost‐effective were 2.3% for UC, 45.3% for AMS, and 52.4% for PSM.

## 4 Discussion

We analyzed the cost‐effectiveness of PSM compared with existing US care models, including UC and AMS. Although DOAC use is increasing, warfarin remains the preferred choice for patients with certain conditions, such as mechanical prosthetic heart valves, moderate‐to‐severe mitral stenosis, antiphospholipid syndrome, and when medication cost is a barrier [56, 57, 58, 59]. In the base‐case analysis, PSM was cost‐saving, delivering better outcomes at lower cost than both UC and AMS. Probabilistic sensitivity analysis supported these findings; across 1000 iterations with varying individual parameters within plausible ranges, most scenarios indicated that PSM was cost‐saving compared with UC (Figure S1A). Additionally, the cost‐effectiveness acceptability curve showed that PSM had the highest probability of being cost‐effective at any willingness‐to‐pay threshold, averaging 52.18% (Figure S2).

A potential explanation for our findings is that PSM may reduce stroke events by approximately half compared with UC and AMS, without increasing bleeding risk. This effect was recently reported in a network meta‐analysis of 8100 patients across 28 trials [8]. Decreasing stroke events is critical because stroke often leads to permanent reductions in quality of life and increased care needs, which translate into long‐term reduction in QALYs and higher associated costs. In our simulated cohort of 10,000 patients (Table S2), PSM reduced stroke events to 1026—about half the number observed with UC (2300) and AMS (2027). This reduction was consistent for both ischemic stroke and intracranial hemorrhage. In contrast, major extracranial hemorrhage events were slightly higher in PSM (2766), compared with UC (2674) and AMS (2395). Most extracranial hemorrhages were non‐fatal, and fatal events were comparable across interventions (UC: 39; AMS: 35; PSM: 41).

**FIGURE 2:**
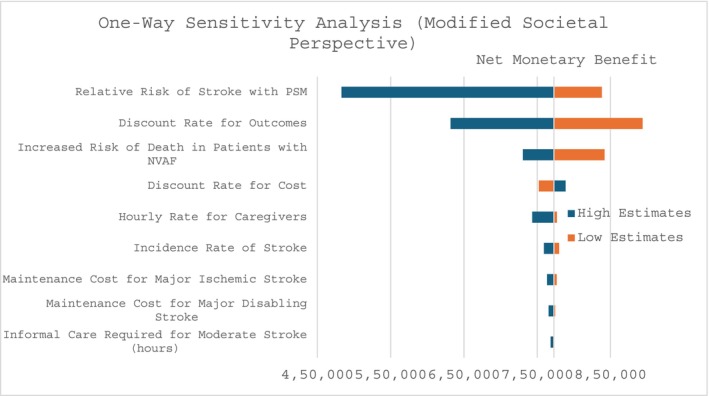
Tornado diagram showing the variation in net monetary benefit when varying between the highest and lowest possible values of each parameter. Abbreviation: NVAF: Non‐valvular atrial fibrillation, PSM: Patient self‐management.

One‐way sensitivity analysis identified the parameters most influential on the NMB of PSM, including the relative risk of stroke in the PSM group, discount rate for outcomes and costs, increased mortality risk in NVAF patients, and the maintenance cost for major ischemic stroke (Figure 2). Stroke is a critical event in NVAF, leading to substantial long‐term costs and disutility; therefore, variations in stroke risks produced the largest impact on NMB. In contrast, PSM‐related costs—such as training and device expenses—are minimal compared with stroke management costs. Consequently, parameters related to stroke incidence and long‐term costs exert the greatest influence on the model. Although some parameters meaningfully affected NMB, the direction of results remained consistent, as indicated by a positive NMB. Because both incremental costs and incremental QALYs were driven primarily by clinical event rates, the cost‐effectiveness acceptability curves were nearly flat and parallel across the willingness‐to‐pay thresholds (Figure S2), confirming that PSM's cost‐effectiveness is robust across plausible parameter ranges and thresholds.

Cost‐effectiveness analyses from other countries consistently show that PSM is cost‐effective compared with other management strategies [18, 60]. In one study, PSM had the highest likelihood of being cost‐effective, with a 78% probability at a willingness‐to‐pay threshold of $5014 per QALY [18]. Another analysis compared PSM with DOACs from an extended Danish health care perspective, incorporating patient‐paid medication costs. PSM was found to be dominant over DOACs, reducing costs while increasing QALYs. Sensitivity analysis further demonstrated that PSM was cost‐effective in 95% of 1000 iterations at a threshold of £20,000 per QALY [60].

The difference in the magnitudes of cost‐effectiveness probabilities is likely explained by variations in relative risks for key events, such as stroke and major extracranial hemorrhage. Prior studies compared PSM only with DOACs, or with DOACs and advanced warfarin care; in particular, the study in Thailand included only UC as the clinic‐based intervention for warfarin [18, 60]. In our analysis, AMS was added as a comparator to better reflect current real‐world practice. Comparing PSM solely with UC produced more pronounced differences in relative risks. In contrast, relative risks for AMS had wider confidence intervals, resulting in greater dispersion in probabilistic sensitivity analyses and a narrower gap in cost‐effective probabilities between AMS and PSM, as shown in the acceptability curve (Figure S2).

No previous US cost‐effectiveness analysis has compared PSM with other anticoagulants or alternative warfarin management strategies. However, several studies have evaluated the cost‐effectiveness of DOACs versus warfarin [25, 28, 34, 61]. Compared with these US analyses, our base‐case results—particularly for UC, fall within a similar range. After adjusting all estimates to the same cost year, published analyses report total costs for warfarin‐treated patients with NVAF ranging from $65,003 to $206,242 and QALYs from 5.87 to 10.28. The discrepancy is primarily driven by the differences in the model structure; specifically, our model does not include “Myocardial Infarction” health state, which is often included in DOACs comparisons. Consequently, a larger portion of our cohort remains in the “Healthy NVAF” state with higher utility. Additionally, variations in input parameters further contribute to the difference in cumulative QALYs.

Several limitations warrant discussion. First, we assumed clinical events were mutually exclusive; once a patient experienced an event—except major extracranial bleeding—they remained in the same health state and could only experience recurrence of that event at a similar or more severe level. In other words, patients did not experience different types of events, which may not reflect real‐world practice. This assumption was necessary due to limitations of Markov modeling, which cannot track individual patient histories. Allowing event types would also disregard the reduced health utility and increased costs following an initial event. Second, we considered ischemic stroke and intracranial hemorrhage as one main stroke event to prevent the overestimation of intracranial hemorrhage cases due to the lack of data on the relative risks across interventions. However, it resulted in reduced flexibility of the model. Third, we assumed intervention effects occurred immediately after initiation and remained constant over time. Long‐term relative risks for PSM remain uncertain, as available trials generally include ≤ 24 months of follow‐up, and effectiveness may attenuate over time. Consistent with standard economic modeling practice, we assumed constant relative risks in the absence of longer‐term empirical data, and we acknowledge this as a limitation of our analysis. These assumptions, made for simplicity and based on data availability, do not fully capture real‐world complexity, where patients may experience multiple events simultaneously, and intervention effects may change over time.

In addition, the model did not explicitly include clinically relevant non‐major bleeding. Although these events do not influence long‐term outcomes, they may increase short‐term health care costs, and their exclusion may underestimate costs in all interventions. We also limited PSM implementation costs to initial training. We acknowledge that potential hidden future costs may vary by setting. Conversely, point‐of‐care INR device costs are likely to decline over time, reducing implementation costs. Although guidelines suggest that some patients may transition from warfarin to DOAC therapy as they age, the long‐term dynamics of such treatment trajectories are uncertain, particularly because phase 3 DOAC atrial fibrillation (AF) trials compared against suboptimally managed warfarin rather than the high‐quality control achievable with PSM. Given the absence of reliable data on switching patterns or time‐varying relative risks, we note this as a limitation and did not model treatment transitions in our base case. Finally, although guidelines recommend DOACs over warfarin for NVAF stroke prevention, a subset of US patients aged 70 years or older cannot afford DOACs due to Medicare coverage limits [62, 63, 64]. In these cases, warfarin remains the preferred anticoagulant.

Appropriate patient selection is essential for safe and effective PSM, and an implementation trial provides the clinical context to support it [65]. In that study, clinicians used structured criteria—clinical stability, cognitive ability or caregiver support, and established home INR monitoring—to identify suitable candidates, followed by a supervised “spotters ready” phase to ensure competency before independent management. Among those selected, 87% successfully transitioned to PSM, time in therapeutic range improved (77.1% to 81.3%), and adverse events did not increase compared with the pre‐PSM period. Clinicians were comfortable with 94% of participants continuing PSM, further demonstrating feasibility when implemented with appropriate safeguards. Our study, therefore, reflects the value of PSM when applied to carefully selected and supported patients rather than the general warfarin‐treated population.

Concerns about the complexity of warfarin dosing are understandable; however, the implementation trial demonstrated that appropriately selected and trained patients can manage PSM safely and effectively [65]. Among participants, TTR improved under PSM compared with clinician‐managed care, and adverse events did not increase during the PSM phase. Notably, patients most often relied on their own treatment experience—rather than dosing support tools—when making adjustments, and this approach was rated as highly useful. Clinician confidence also increased over time, with 94% comfortable having their patients continue PSM at study end. These findings suggest that, when limited to stable and experienced patients, PSM is both feasible and clinically sound despite the perceived complexity of warfarin management.

Prior studies have identified characteristics associated with poorer self‐management in some populations, underscoring the importance of structured patient selection and training when implementing PSM. In the implementation trial, these safeguards—including assessment of clinical stability, cognitive readiness, and home INR monitoring experience—allowed even older adults to perform PSM safely with improved TTR and no increase in adverse events [65].

Although some older adults with multimorbidity may benefit from more frequent clinical contact, routine INR visits generally address anticoagulation only and do not replace primary care. Importantly, PSM does not limit patients' ability to seek care as needed, and in the implementation trial, PSM participants did not experience higher rates of emergency visits or hospitalizations than in the pre‐PSM period [65]. Frequent clinic or laboratory visits can also be particularly burdensome for rural patients and those with limited transportation or mobility. Thus, although our model does not capture potential indirect benefits of additional clinician contact, these must be balanced against the burden of frequent visits and the demonstrated safety of PSM among appropriately selected individuals.

Overall, PSM demonstrated dominance by improving outcomes and reducing costs, primarily through lowering stroke risk, which in turn reduces long‐term care costs. In addition, PSM enhances patient satisfaction and reduces healthcare provider workload. Therefore, PSM should be considered for broader implementation in US health settings. Although anticoagulation therapy is trending toward DOACs, warfarin remains the critical alternative for patients with mechanical heart valves, valvular atrial fibrillation, antiphospholipid syndrome, and for those who prefer warfarin for non‐clinical reasons, including cost considerations [66, 67]. PSM is thus a valuable strategy for optimizing warfarin management within the US health care system.

## Author Contributions

**Warittakorn Kategeaw:** writing – original draft, formal analysis, methodology. **Daniel M. Witt:** conceptualization, supervision, funding acquisition, writing – review and editing, validation. **Jordan B. King:** conceptualization, validation, writing – review and editing. **Daniel C. Malone:** supervision, writing – review and editing, validation. **Nathorn Chaiyakunapruk:** conceptualization, methodology, supervision, validation, writing – review and editing.

## Funding

This study received financial support from the Agency for Healthcare Research and Quality under the main project titled “Overcoming Barriers to Warfarin Patients Self‐Management in the US Healthcare System” (grant ID 5 R18HS027960).

## Conflicts of Interest

The authors declare no conflicts of interest.

## Supporting information


**Figure S1:**. (A) Probabilistic sensitivity analysis illustrating possible incremental cost (y‐axis) corresponding with incremental QALY (x‐axis) when varying individual parameters within their possible ranges, given the assumed distribution, simultaneously.
(B) Probabilistic sensitivity analysis illustrating possible incremental cost (y‐axis) corresponding with incremental QALY (x‐axis) when varying individual parameters within their possible ranges, given the assumed distribution, simultaneously, comparing AMS with UC.
(C) Probabilistic sensitivity analysis illustrating possible incremental cost (y‐axis) corresponding with incremental QALY (x‐axis) when varying individual parameters within their possible ranges, given the assumed distribution, simultaneously, comparing PSM with UC.
(D) Probabilistic sensitivity analysis illustrating possible incremental cost (y‐axis) corresponding with incremental QALY (x‐axis) when varying individual parameters within their possible ranges, given the assumed distribution, simultaneously, comparing PSM with AMS.
**Figure S2:** Probabilistic sensitivity analyses revealed that the probability of PSM being cost‐effective over a plausible range of willingness‐to‐pay thresholds.
**Table S1:** CHEERS 2022 Checklist.
**Table S2:** Number of events per 10,000 people over 30 years.

## Data Availability

All data supporting the findings are reported within the article and/or Supporting Information. Data sharing is not applicable to this article as no datasets were generated or analyzed during the current study.
