# Supplementary material for: Cost‐Effectiveness Analysis of Patient Self‐Management of Warfarin Among Patients With Non‐Valvular Atrial Fibrillation
Source: Pharmacotherapy. 2026 May 14;46:e70154. doi: 10.1002/phar.70154 (PMC13176797; doi:10.1002/phar.70154)
Supplement: Supplementary file 1 — Figure S1:. (A) Probabilistic sensitivity analysis illustrating possible incremental cost (y‐axis) corresponding with incremental QALY (x‐axis) when varying individual parameters within their possible ranges, given the assumed distribution, simultaneously. (B) Probabilistic sensitivity analysis illustrating possible incremental cost (y‐axis) corresponding with incremental QALY (x‐axis) when varying individual parameters within their possible ranges, given the assumed distribution, simultaneously, comparing AMS with UC. (C) Probabilistic sensitivity analysis illustrating possible incremental cost (y‐axis) corresponding with incremental QALY (x‐axis) when varying individual parameters within their possible ranges, given the assumed distribution, simultaneously, comparing PSM with UC. (D) Probabilistic sensitivity analysis illustrating possible incremental cost (y‐axis) corresponding with incremental QALY (x‐axis) when varying individual parameters within their possible ranges, given the assumed distribution, simultaneously, comparing PSM with AMS. Figure S2: Probabilistic sensitivity analyses revealed that the probability of PSM being cost‐effective over a plausible range of willingness‐to‐pay thresholds. Table S1: CHEERS 2022 Checklist. Table S2: Number of events per 10,000 people over 30 years. [file PHAR-46-0-s001.docx]

**Supplementary Materials**

**Supplement Table 1.** CHEERS 2022 Checklist

**Supplement Table 2.** Number of events per 10,000 people over 30 years

**Supplement Figure 1A.** Probabilistic sensitivity analysis illustrating possible incremental cost (y-axis) corresponding with incremental QALY (x-axis) when varying individual parameters within their possible ranges, given the assumed distribution, simultaneously

**Supplement Figure 1B.** Probabilistic sensitivity analysis illustrating possible incremental cost (y-axis) corresponding with incremental QALY (x-axis) when varying individual parameters within their possible ranges, given the assumed distribution, simultaneously, comparing AMS with UC

**Supplement Figure 1C.** Probabilistic sensitivity analysis illustrating possible incremental cost (y-axis) corresponding with incremental QALY (x-axis) when varying individual parameters within their possible ranges, given the assumed distribution, simultaneously, comparing PSM with UC

**Supplement Figure 1D.** Probabilistic sensitivity analysis illustrating possible incremental cost (y-axis) corresponding with incremental QALY (x-axis) when varying individual parameters within their possible ranges, given the assumed distribution, simultaneously, comparing PSM with AMS

**Supplement Figure 2.** Probabilistic sensitivity analyses revealed that the probability of PSM being cost-effective over a plausible range of willingness-to-pay thresholds

**Supplement Table 1.** CHEERS 2022 Checklist

| **Topic** | **No.** | **Item** | **Location where item is reported** |
| --- | --- | --- | --- |
| **Title** |  |  |  |
|  | 1 | Identify the study as an economic evaluation and specify the interventions being compared. | Page 1 |
| **Abstract** |  |  |  |
|  | 2 | Provide a structured summary that highlights context, key methods, results, and alternative analyses. | Page 2 |
| **Introduction** |  |  |  |
| **Background and objectives** | 3 | Give the context for the study, the study question, and its practical relevance for decision making in policy or practice. | Page 3 |
| **Methods** |  |  |  |
| **Health economic analysis plan** | 4 | Indicate whether a health economic analysis plan was developed and where available. | Page 4 |
| **Study population** | 5 | Describe characteristics of the study population (such as age range, demographics, socioeconomic, or clinical characteristics). | Page 4 |
| **Setting and location** | 6 | Provide relevant contextual information that may influence findings. | Page 4 |
| **Comparators** | 7 | Describe the interventions or strategies being compared and why chosen. | Page 4 |
| **Perspective** | 8 | State the perspective(s) adopted by the study and why chosen. | Page 4 and 6 |
| **Time horizon** | 9 | State the time horizon for the study and why appropriate. | Page 4 and 6 |
| **Discount rate** | 10 | Report the discount rate(s) and reason chosen. | Page 6 |
| **Selection of outcomes** | 11 | Describe what outcomes were used as the measure(s) of benefit(s) and harm(s). | Page 6 |
| **Measurement of outcomes** | 12 | Describe how outcomes used to capture benefit(s) and harm(s) were measured. | Page 6 |
| **Valuation of outcomes** | 13 | Describe the population and methods used to measure and value outcomes. | Page 4 and 6 |
| **Measurement and valuation of resources and costs** | 14 | Describe how costs were valued. | Page 5-6 |
| **Currency, price date, and conversion** | 15 | Report the dates of the estimated resource quantities and unit costs, plus the currency and year of conversion. | Page 5-6 |
| **Rationale and description of model** | 16 | If modelling is used, describe in detail and why used. Report if the model is publicly available and where it can be accessed. | Page 4 and 6 |
| **Analytics and assumptions** | 17 | Describe any methods for analysing or statistically transforming data, any extrapolation methods, and approaches for validating any model used. | Page 4-6 |
| **Characterising heterogeneity** | 18 | Describe any methods used for estimating how the results of the study vary for subgroups. | NA |
| **Characterising distributional effects** | 19 | Describe how impacts are distributed across different individuals or adjustments made to reflect priority populations. | NA |
| **Characterising uncertainty** | 20 | Describe methods to characterise any sources of uncertainty in the analysis. | Page 6 |
| **Approach to engagement with patients and others affected by the study** | 21 | Describe any approaches to engage patients or service recipients, the general public, communities, or stakeholders (such as clinicians or payers) in the design of the study. | Page 4 |
| **Results** |  |  |  |
| **Study parameters** | 22 | Report all analytic inputs (such as values, ranges, references) including uncertainty or distributional assumptions. | Table 1 |
| **Summary of main results** | 23 | Report the mean values for the main categories of costs and outcomes of interest and summarise them in the most appropriate overall measure. | Page 6-7 and Table 2 |
| **Effect of uncertainty** | 24 | Describe how uncertainty about analytic judgments, inputs, or projections affect findings. Report the effect of choice of discount rate and time horizon, if applicable. | Figure 2, Supplement Figure 1-2 |
| **Effect of engagement with patients and others affected by the study** | 25 | Report on any difference patient/service recipient, general public, community, or stakeholder involvement made to the approach or findings of the study | Page 4 |
| **Discussion** |  |  |  |
| **Study findings, limitations, generalisability, and current knowledge** | 26 | Report key findings, limitations, ethical or equity considerations not captured, and how these could affect patients, policy, or practice. | Page 7-10 |
| **Other relevant information** |  |  |  |
| **Source of funding** | 27 | Describe how the study was funded and any role of the funder in the identification, design, conduct, and reporting of the analysis | Page 11 |
| **Conflicts of interest** | 28 | Report authors conflicts of interest according to journal or International Committee of Medical Journal Editors requirements. | Page 11 |

*From:* Husereau D, Drummond M, Augustovski F, et al. Consolidated Health Economic Evaluation Reporting Standards 2022 (CHEERS 2022) Explanation and Elaboration: A Report of the ISPOR CHEERS II Good Practices Task Force. Value Health 2022;25. [doi: 10.1016/j.jval.2021.10.008](doi:10.1016/j.jval.2021.10.008)

**Supplement Table 2.** Number of events per 10,000 people over 30 years

| Event | Number of events (95% Confidence Interval) | | |
| --- | --- | --- | --- |
|  | Usual Care | Anticoagulation  Management Services | Patient Self-Management |
| **Total Stroke** (Ischemic stroke and Intracranial hemorrhage) | 2,300 (1,984, 2,648) | 2,027 (73, 8,175) | 1,026 (126, 3,287) |
|  |  |  |  |
| **Ischemic Stroke** | | | |
| Total events | 2,059 (1,769, 2,384) | 1,784 (64, 7,312) | 897 (110, 2,899) |
| Minor | 792 (692, 898) | 688 (28, 2055) | 364 (47, 1,081) |
| Major | 810 (687, 952) | 689 (22, 2,952) | 336 (40, 1,128) |
| Disabled | 279 (239, 322) | 260 (8, 1,395) | 117 (14, 391) |
| Fatal | 179 (152, 210) | 168 (5, 901) | 80 (9, 300) |
| **Intracranial Hemorrhage** | |  |  |
| Total events | 241 (212, 271) | 242 (9, 854) | 129 (16, 382) |
| Minor | 48 (42, 54) | 47 (2, 154) | 25 (3, 72) |
| Major | 39 (34, 44) | 37 (1, 126) | 20 (3, 58) |
| Disabled | 27 (23, 30) | 25 (1, 91) | 14 (2, 39) |
| Fatal | 128 (112, 144) | 133 (5, 484) | 70 (9, 212) |
| **Major Extracranial Hemorrhage** | |  |  |
| Total events | 2,674 (2,236) | 2,395 (1,199, 4,121) | 2,766 (1,812, 3,993) |
| Non-Fatal | 2,635 (2,203, 3,076) | 2,360 (1,182, 4,060) | 2,725 (1,785, 3,935) |
| Fatal | 39 (33, 46) | 35 (18, 61) | 41 (27, 59) |

**Supplement Figure 1A.** Probabilistic sensitivity analysis illustrating possible incremental cost (y-axis) corresponding with incremental QALY (x-axis) when varying individual parameters within their possible ranges, given the assumed distribution, simultaneously

**Abbreviation**

**AMS** = Anticoagulation Management Services; **PSM** = Patient Self-Management; **QALY** = Quality-Adjusted Life Years; **UC** = Usual Care

**Supplement Figure 1B.** Probabilistic sensitivity analysis illustrating possible incremental cost (y-axis) corresponding with incremental QALY (x-axis) when varying individual parameters within their possible ranges, given the assumed distribution, simultaneously, comparing AMS with UC

**Abbreviation**

**AMS** = Anticoagulation Management Services; **QALY** = Quality-Adjusted Life Years; **UC** = Usual Care

**Supplement Figure 1C.** Probabilistic sensitivity analysis illustrating possible incremental cost (y-axis) corresponding with incremental QALY (x-axis) when varying individual parameters within their possible ranges, given the assumed distribution, simultaneously, comparing PSM with UC

**Abbreviation**

**PSM** = Patient Self-Management; **QALY** = Quality-Adjusted Life Years; **UC** = Usual Care

**Supplement Figure 1D.** Probabilistic sensitivity analysis illustrating possible incremental cost (y-axis) corresponding with incremental QALY (x-axis) when varying individual parameters within their possible ranges, given the assumed distribution, simultaneously, comparing PSM with AMS

**Abbreviation**

**AMS**  = Anticoagulation Management Services; **PSM** = Patient Self-Management; **QALY** = Quality-Adjusted Life Years

**Supplement Figure 2.** Probabilistic sensitivity analyses revealed the probability of PSM being cost-effective over a plausible range of willingness-to-pay thresholds

**Abbreviation**

**AMS** = Anticoagulation Management Services; **PSM** = Patient Self-Management; **UC** = Usual Care
